# Ability of the LACE Index to Predict 30-Day Readmissions in Patients with Acute Myocardial Infarction

**DOI:** 10.3390/jpm12071085

**Published:** 2022-06-30

**Authors:** Vasuki Rajaguru, Tae Hyun Kim, Jaeyong Shin, Sang Gyu Lee, Whiejong Han

**Affiliations:** 1 Department of Healthcare Management, Graduate School of Public Health, Yonsei University, Seoul 03722, Korea; vasuki@yuhs.ac (V.R.); thkim@yuhs.ac (T.H.K.); 2 Department of Preventive Medicine, College of Medicine, Yonsei University, Seoul 03722, Korea; drshin@yuhs.ac (J.S.); leevan@yuhs.ac (S.G.L.); 3 Department of Global Health Security, Graduate School of Public Health, Yonsei University, Seoul 03722, Korea

**Keywords:** readmission, acute myocardial infarction, risk assessment, prediction

## Abstract

Aims: This study aimed to utilize the existing LACE index (length of stay, acuity of admission, comorbidity index and emergency room visit in the past six months) to predict the risk of 30-day readmission and to find the associated factors in patients with AMI. Methods: This was a retrospective study and LACE index scores were calculated for patients admitted with AMI between 2015 and 2019. Data were utilized from the hospital’s electronic medical record. Multivariate logistic regression was performed to find the association between covariates and 30-day readmission. The risk prediction ability of the LACE index for 30-day readmission was analyzed by receiver operating characteristic curves with the C statistic. Results: A total of 205 (5.7%) patients were readmitted within 30 days. The odds ratio of older age group (OR = 1.78, 95% CI: 1.54–2.05), admission via emergency ward (OR = 1.45; 95% CI: 1.42–1.54) and LACE score ≥10 (OR = 2.71; 95% CI: 1.03–4.37) were highly associated with 30-day readmissions and statistically significant. The receiver operating characteristic curve C statistic of the LACE index for AMI patients was 0.78 (95% CI: 0.75–0.80) and showed favorable discrimination in the prediction of 30-day readmission. Conclusion: The LACE index showed a good discrimination to predict the risk of 30-day readmission for hospitalized patients with AMI. Further study would be recommended to focus on additional factors that can be used to predict the risk of 30-day readmission; this should be considered to improve the model performance of the LACE index for other acute conditions by using the national-based administrative data.

## 1. Introduction

Readmission frequency is used to judge hospital quality as 30 days of unplanned readmission indicates the initial intervention was unsuccessful, and hospital readmissions result in $17 billion in annual medical costs, according to the Centers for Medicare and Medicaid Services (CMS) [1]. As part of the 2010 Hospital Readmission Reduction Program, CMS classified chronic diseases with a high risk of frequent hospitalization [1,2]. In general, cardiovascular diseases (CVDs) are considered a leading cause of unexpected mortality and morbidity and a serious public health concern globally [2].

Acute myocardial infarction (AMI) is a critical global health issue which causes more than seven-million deaths worldwide per year [3]. According to the evaluation of the Healthcare Cost and Utilization Project (HCUP), AMI would have an unplanned readmission during the first 30 days of hospital discharge, which has an estimated direct cost of $1 billion in annual Medicare expenditures in the United States [4]. Statistically, nearly 20% of Medicare beneficiaries were readmitted within 30 days after an AMI [3,4]. Payment incentives and Medicare hospital readmission penalties were developed to reduce the 30-day readmission rates [2]. A number of hospitals across the nation have implemented strategies aimed at reducing avoidable hospital-related complications, improving the standard of patient care, and improving communication and care coordination with other healthcare providers to produce relatively low readmission rates. [4,5]. As a result, policymakers and healthcare professionals have focused on reducing readmission rates as a strategy for enhancing treatment quality and reducing healthcare spending [5]. Therefore, the readmission rates are publicly reported, and recent health-reform legislation endorsed the use of readmission rates for hospital profiling in various countries [6].

Some studies have been undertaken to reduce AMI-related unplanned 30-day readmissions; however, those results and guidelines could not be recommended widely [4,7,8,9]. The widely accepted common characteristic of AMI is that it is difficult to cure once it has developed due to the structural dysfunction that cannot be fixed [10]. It may lead to serious complications, requiring follow-up visits to medical facilities and repeated readmissions, including 30-day readmissions in acute care setting in patients with cardiovascular diseases, which are potentially avoidable [11,12].

The prediction of the risk of a 30-day readmission has been developed using various tools and models [13,14,15]. Most of them have limited generalizability and cannot be applied to other healthcare systems due to unique variables in their specific settings. The LACE index is one of the most commonly used indices in the US and Canada [16,17,18,19,20,21,22]. It was first developed by van Walraven et al. [21] to predict the risk of unplanned readmission or death within 30 days after hospital discharge in medical and surgical patients. The model includes the length of hospitalization stay (L), acuity of the admission (A), comorbidities of patients (C) and the number of emergency department visits in the six months before admission (E). Scores range from 0 to 19 and the higher scores indicate a high risk of readmission, with scores greater than 10 considered high likely for a 30-day readmission [23]. This tool is widely used, primarily because its simplicity makes it suited to day-to-day clinical practice [17,18,19,20,21,22,23,24,25].

Numerous studies have created models using the LACE index to predict a high risk of 30-day readmission. The literature on risk prediction of 30-day readmission emphasizes small patient populations [22,23,24,25] or specific patient groups, such as those suffering from cardiovascular disease [18,19,20,22]. Very little is known about the LACE index in Asian countries [23,24], and no study related to the use of the LACE index has been conducted in South Korea. Risk prediction of 30-day readmission for patients with AMI could be accomplished with a variety of assessment tools ranging from multidisciplinary patient interviews to simple screening tools using a handful of variables [26,27,28]. Several studies have investigated predictors: demographic characteristics, admission and discharge types, comorbidities, length of stay, medications and special procedures associated with 30-day readmissions [29,30]. However, healthcare professionals still struggle to predict those patients who are at high risk of hospital readmission. To be able to do so for patients hospitalized with AMI would be helpful as it would enable targeted interventions.

Therefore, we have conducted a systematic review and meta-analysis study on the LACE index to predict 30-day readmission, and we have identified 16 studies [31]. Our systematic review found a comparable ability to predict 30-day readmission by using the LACE index for the patients admitted with cardiopulmonary diseases in an acute care setting. Most of them were carried out in heart failure patients and all-cause readmissions; none of them have focused on AMI. In that context, this study utilized the LACE index to predict the risk of 30-day readmissions in AMI patients after discharge from hospital, which no prior study had carried out in South Korea. Further study will continue to assess the model’s performance by comparing the risk prediction ability against 60-day, 90-day and one-year hospital readmissions.

## 2. Materials and Methods

### 2.1. Study Design and Setting

A retrospective cohort study design was adopted, and data from January 2015 to December 2019 were collected from the electronic health record data of a single university-affiliated hospital in Seoul, South Korea. Patients aged 19 years and over were eligible if they were hospitalized for AMI as the principal diagnosis with an International Classification of Disease, 10 (ICD-10) code (I20-I25). Admissions were not considered as an index admission if the patients were <18 years old, transferred to another acute care hospital, or had missing data. AMI patients who died after the 30-day readmission and patients who had at least one readmission within 30 days for respective 30-day readmission analysis were included in our study. Time to readmission was calculated by subtracting the length of stay of index admissions from the time between the two admissions. However, only the first readmission and respective index readmission were included in the final analysis.

This study protocol was reviewed and approved by the Institutional Review Committee of Yonsei University (4-2021-1047-001, date of approval: 8 September 2021). After ethical consideration, the need for patient consent was waived, and confidentiality was ensured by de-identifying all data that were potentially identifiable.

### 2.2. Dependent Variables

Our primary outcome was defined as hospital readmission within 30 days for patients diagnosed with any type of AMI as an index of hospitalization. The LACE index score was calculated for each patient based on their length of stay (L), acuity of admission (A), comorbidities (C) and emergency visits within the past six months. The scoring patterns were calculated and reported in previous studies [16,17]. The length of stay was calculated from the first to the last day of hospitalization, and patients admitted to the hospital via the emergency department were identified as having an acuity of admission. Comorbidities were measured on the Charlson comorbidity index (CCI) and the International Classification of Diseases, 10 (ICD-10). Emergency visits in the past six months were measured, with multiple emergency visits within 24 h considered as one visit.

### 2.3. Independent Variables

The demographic data included a patient’s age, sex, residence, and insurance type. The age of AMI patients was divided into three groups, sex was male or female and insurance type was specified (e.g., national health insurance, Medicare). We considered three indexes of admission: via the emergency department, as an outpatient or transferred from another hospital. There were two discharge types: normal (those who were discharged with a full recovery) and “other” (discharged with the necessary preventive measures or against medical advice). The discharge destination was classified into two types: normal (heading home with a full recovery) and a transfer to another hospital or nursing home, or facility. In addition, the length of stay (LoS), comorbidities by ICD-10 code, primary diagnosis, treatment specialty, admission source and discharge destination were obtained from the hospital EMR data. The 30-day readmission was tracked to identify patients’ discharge and readmission history, and this report was manually confirmed through a chart review.

### 2.4. Data Analysis

The data were analyzed in three ways. First, we performed chi-squared and univariate comparisons between a 30-day readmission and no-readmission; the mean (M) and standard deviation (SD) were used for continuous data, and frequency and percentage were used for categorical data. Second, a multivariate logistic regression analysis was carried out to find the factors associated with 30-day readmissions, based on the odds ratio (OR; 95% CI). Third, the LACE index score was calculated for an individual patient. We created ROC curves to assess the sensitivity and specificity of the risk prediction model. In addition, the C-statistic was used to evaluate the discrimination ability of the model, which ranged from 0.5 (low discrimination) to 1 (good/high discrimination), as measured by the area under the curve (AUC). Finally, we determined a suitable numerical threshold by fitting a logistic regression model for each outcome to LACE scores above and below specific thresholds using ORs (95% CI), while the Kaplan–Meier survival analysis with a log-rank test was used to compare the LACE score with 30-day readmission data. Two-tailed *p*-values < 0.05 were considered to indicate statistical significance. Statistical analysis was conducted using SAS 9.4 (SAS Institute Inc., Cary, NC, USA).

## 3. Results

### 3.1. Study Population and Characteristics

**Figure 1:**
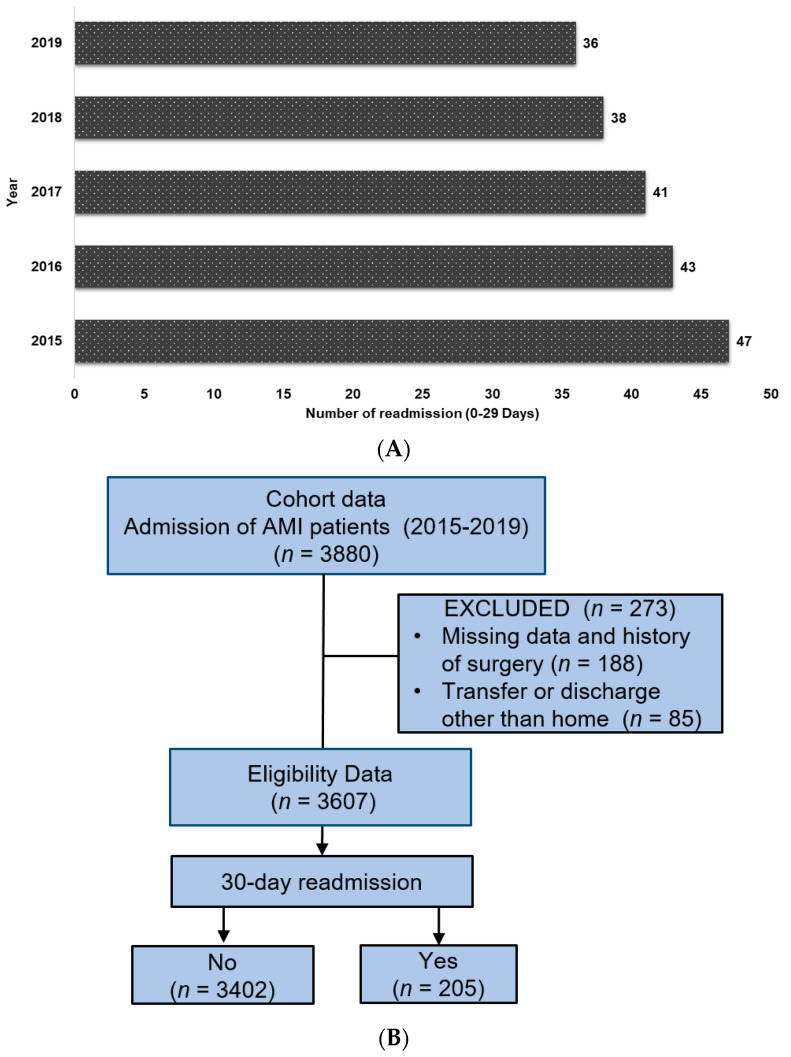
(**A**). Trends in 30-day acute myocardial infarction (AMI)-specific readmissions between 2015 and 2019. (**B**). Flow chart for selection process of study population.

The study cohort included a total of 3880 patients with AMI; of these, 3607 patients were deemed eligible for the final analysis after excluding the data of death. For 205 (5.7%) of those patients, 30-day readmissions were documented, and 30-day readmission trends between 2015 and 2019 showing a gradual decrease among patients hospitalized with AMI was observed (Figure 1A,B).

### 3.2. Characteristics of 30-Day Readmissions versus Non-Readmitted Patients

Table 1 summarizes the observed frequency (percentage) and mean (standard deviation) baseline data of 30-day readmissions and non-readmissions. Over half of the patients were male (120, 58.5%) and the average mean age was 68 years (68.4 (12.9)). Most of the patients resided in Seoul (158, 77.1%) and had national health insurance membership (114, 55.6%). The length of stay (LOS) was about three days (81, 39.5%), and those admitted via an emergency department visit (106, 51.7%) or with three and more comorbidities (78, 38%) showed a higher percentage of 30-day readmissions. Laboratory findings showed patients admitted within 30 days had significantly lower hemoglobin levels (10.6 ± 9.3; *p* < 0.001); however, there were no statistical differences in any other laboratory findings.

Table 2 illustrates the observed frequency (percentage) and mean (standard deviation) baseline data of 30-day readmissions according to the LACE index score. Over half of the patients were male (54, 58.7%) with an average age of 68 years. The length of stay (LOS) was approximately three days (34, 47.9%), and those with scores of 5–9 showed higher 30-day readmissions than those with other scores. The index of admission via an emergency department visit, with a LACE index score of 0–4, was higher (30, 71.4%); those with more than three comorbidities (19, 45.2%) and who had more than 10 emergency visits over the past 6 months (45, 48.9%) showed higher 30-day readmissions.

### 3.3. Association between the Risk Factors and 30-Day Readmissions of AMI Patients

Following a multivariate logistic regression analysis, the risk factors determined to be independently associated with 30-day readmissions are shown in Table 3. The older patients aged ≥65 years (OR, 8.15; 95% CI, 4.07–6.24) had a higher association than other age groups. In addition, men (OR, 1.07; 95% CI, 1.06–1.07), those with a Medicare insurance membership (OR, 1.07; 95% CI, 1.00–1.11) and those who were readmitted via the emergency route (OR, 1.45; 95% CI, 1.42–1.54) were highly associated with a 30-day admission. In terms of the discharge type, “other” (OR, 1.09; 95% CI, 1.04–1.14; as opposed to normal) showed a higher association that was statistically significant after controlling potential confounders; the same was true in terms of the discharge destination being transfer to another hospital/facility (OR, 1.96; 95% CI, 1.15–3.33). In addition, LACE index risk scores (OR, 2.71; 95% CI, 1.03–4.37) were highly associated with other risk scores.

Associations between the different LACE variables were found to highly predict the risk of a 30-day readmission. The length of stay (OR, 2.01; 95% CI, 1.35–2.98), index of admission (OR, 1.21; 95% CI, 1.01–1.44), comorbidities (OR, 1.72; 95% CI, 1.16–2.55) and number of emergency visits in the last six months (OR, 1.61; 95% CI, 1.14–2.52) were statistically significant at the *p* < 0.001 level.

### 3.4. Sensitivity Analysis

**Figure 2:**
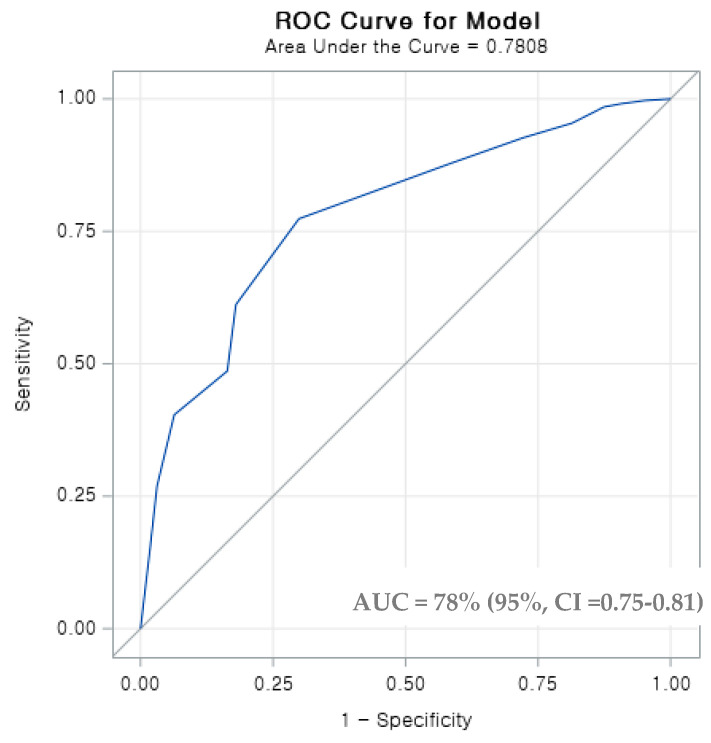
Receiver operating characteristic (ROC) curve for the LACE index in hospitalized AMI patients. The ROC curve illustrates the LACE index predictions of a high risk of 30-day readmission at different cut-off points with increased sensitivity and decreased specificity. The area under the curve (AUC), which is equal to the C-statistic (0.78), indicates a favorable model to predict the risk of a 30-day readmission in patients with AMI.

The discrimination ability of the model for risk prediction of 30-day readmission, in Figure 2, shows a modest performance of the LACE index with a C-statistic of 0.78 (95% CI 0.75–0.81). The ROC analysis outcome for a 30-day readmission is shown in an AUC (Figure 2). These findings indicate that the LACE index model has a favorable risk prediction ability for the 30-day readmission of patients hospitalized with AMI.

### 3.5. Survival Analysis

**Figure 3:**
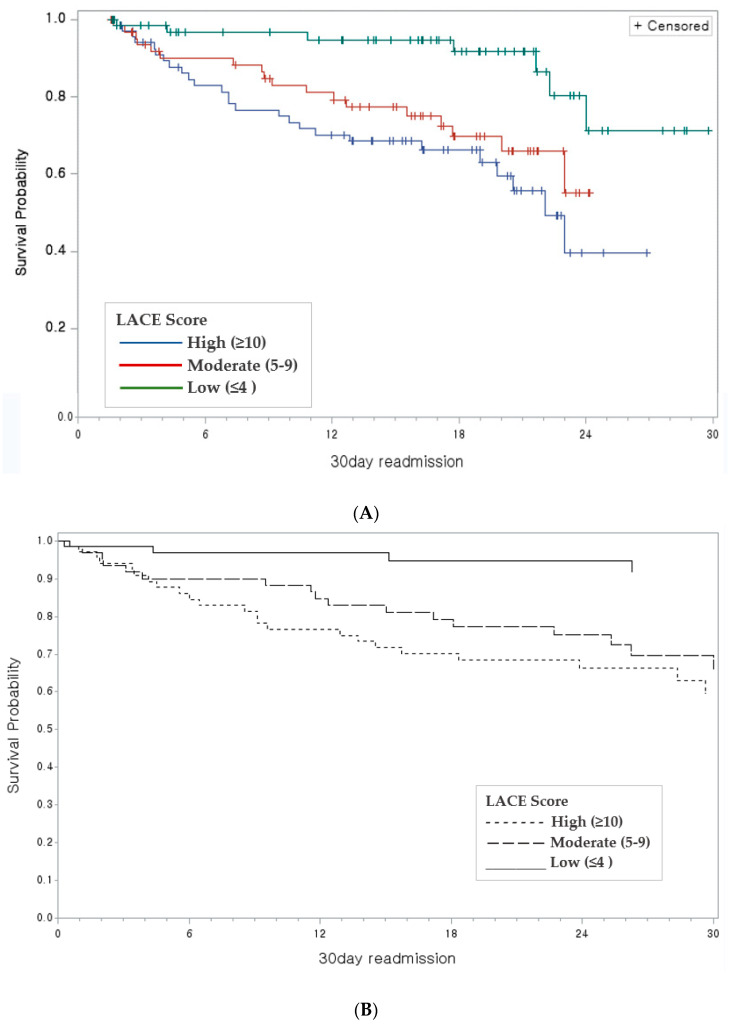
Kaplan–Meier survival plot: (**A**). Product limit estimates of survival of for patients with AMI and colors indicates the LACE scores; (**B**) Overall survival of AMI patients with 30-day readmission and LACE index scores <4 (black line), 5–9 (dashed blue line) and ≥10 (solid dashed line).

According to the Kaplan–Meier curves illustrated in Figure 3A,B, a LACE score below 4 had a lower readmission likelihood than a moderate (5–9) or high score (>10). The log-rank test showed a statistically significant (*p* < 0.05) higher likelihood of a 30-day readmission in patients with AMI.

## 4. Discussion

This study aimed to predict a high risk of 30-day readmission by using the LACE index score and validated models for patients hospitalized with AMI. A systematic review of 16 unique LACE index articles was used as the basis to predict the risk of a 30-day readmission in a specific disease and among the population of one country [31]; no such studies had previously been carried out in South Korea. A single hospital retrospective study indicates that the LACE score has good discrimination and calibration to predict 30-day readmissions for patient hospitalized with AMI.

The overall readmission rate was lower than the reported rate by 15.5~15.9% [7,26,27]. However, it is difficult to compare studies directly because the published studies used Medicare’s fee-for-service claims data in the US and included only elderly Medicare patients. Our study found men were 13% more likely to have a 30-day readmission than women. This finding is similar to that of an earlier retrospective study conducted on patients with heart failure or COPD, where all-cause readmissions were predicted using the LACE index. Our study population had a high Charlson comorbidity index score, resulting in a higher association and significance with a 30-day readmission (*p* < 0.001). Patients with more comorbidities were more likely to be readmitted to the hospital, especially when one or more of these serious conditions occurred during the index hospitalization. Our results support those of previous studies that have demonstrated associations between older age and multi comorbidities [18,22,25]. However, the population examined when the LACE index was initially derived had a score much lower than that of our study population.

Our study found higher 30-day readmission rates among men, those aged 65 years and older, patients from lower-income households, Medicare beneficiaries, and those who had multiple comorbidities, and similar results were reported on CVD-related 30-day readmission rates [12,18,19,20,26,27,30]. We also found that patients who were discharged for other reasons, such as those discharged against medical advice or voluntarily discharged, were more likely to have 30-day readmissions compared to those who had a normal discharge. This finding is consistent with other studies [8,9,10,11,12], and the LACE index found varying disease conditions [16,17,18,19]. Therefore, interventions are required to improve the after-discharge support for patients who are discharged to other facilities such as nursing homes, which will be helpful to prevent or reduce readmissions.

A significant finding of this study was that 30-day readmissions were related to sociodemographic factors rather than clinical findings on the index of admission, though this could not be considered an important risk factor to predict the 30-day readmission since important clinical variables were not tested. This approach was consistent with existing studies on different disease conditions where the clinical findings had barely or not been considered [5,8,10,11,12,14,16,17,18,19,27]. It may be difficult to deploy clinical findings from laboratory results due to their complexity and possible overfitting. This is consistent with how LACE has performed in older American, British and Australian populations [16,17,19,20]. Moreover, Zhang et al., reported that ultrasonic examinations, laboratory tests and detailed real clinical data were applied to find the risk predictionand it made it more convenient to predict the 30-day readmission risk [7]. However, this study was not focused LACE index. Nevertheless, the findings so far on the prediction of readmission for acute care suggest that attention should be paid to clinical findings in long-term care, more so than in acute care settings.

Hence, we speculate that fluctuations in emergency room visit trends could be a cause of the variations in readmissions among emergency cardiovascular conditions observed in the later years of the study period. Studies have reported various factors that contribute to a 30-day readmission, including complications of inpatient treatment, poor coordination of care, low quality of care and ineffective medication advice, discharge education or follow-up [8,28,29]. In contrast to the LACE index, the length of stay and acuity of admission were not associated with the risk of 30-day readmission after adjusting covariates in the multivariate logistic regression model. It could be possible, but the duration of admission was affected by other factors such as demographic characteristics and did not reflect the severity of illness entirely in this cohort study.

Our previous literature review identified that many other factors were significant in the risk prediction of 30-day readmissions such as age, comorbidity index and emergency department visits in the past six months [31]. Most of the predictive models showed a statistically significant difference in patients, with chronic readmissions among patients with cardiovascular diseases [18,19,20,21,22,29]. Our findings revealed that patients with a LACE score above 10 had an absolute possibility of a 30-day readmission, and patients with Medicare insurance had similar results. Implementing the LACE index has been associated with a decrease in cardiovascular disease readmissions. Some studies have reported a low or poor ability to predict 30-day readmissions after the LACE index’s implementation [18,19,20,21,22,23,24,25].

Our results on the predictive ability of the model for the 30-day readmission risk for hospitalized AMI patients had a C-statistic of 0.78, range of 0.75–0.8. Thus, the predictive ability of our model was slightly better than those of other commonly cited readmission risk-prediction models [16,17,18,20,22,25]. When we evaluated its performance, the discrimination ability was favorable to predict risk of a 30-day readmission when compared to our derived model (C-statistic of 0.65) in the Canadian population from which it was derived (C-statistic of 0.69) [21]. Other studies focused on a predictive model that looked at 30-day readmissions in patients with different disease conditions, ranging from 0.56 to 7.2. They found the rates of 30-day readmission to be low or similar to our findings in a previous systematic review [31]. Low et al. reported a similar predictive ability of the C-statistic (0.78, 95% CI: 0.77–0.79) for the 30-day readmission risk among pneumonia patients [23]. However, in patients with heart failure, there was disagreement or a lower discrimination ability (0.57 [18], 0.67 [22] or 0.56 [20]) in the USA. The variation in the predictive ability of the C-statistic model of LACE index scores could be based on the quantity of data or risk factors. Patients in the Canadian cohort were mainly free of serious comorbidities, while our AMI cohort had higher CCI scores [21]. The difference in performance is not surprising as the cohort enrolled in the Canadian study held a lower risk profile and differed from the usual patients who attend AMI wards in South Korea.

The LACE index allows clinicians to calculate an individual’s unique risk of a 30-day readmission quickly and accurately. In addition, enabling improved coordination of care between healthcare professionals and the implementation of various strategies could prevent readmissions among high-risk patients. Reducing readmissions not only reduces healthcare expenditure but also, most importantly, improves patient outcomes and satisfaction. Readmissions are not only inconvenient and costly for the patient but also come with inherent risks such as hospital-acquired infections, which impact negatively on patient outcomes. Therefore, this study suggests using the LACE index as it can be helpful for physicians to make better clinical decisions about the duration and aggressiveness of patient treatment and management and may help curtail premature discharges for patients with a high readmission risk.

The strengths of our study were the data over a five-year study period, which allowed us to analyze trends in 30-day readmission rates over five years. Furthermore, we benefitted from access to data on the length of stay from the original data, which was very helpful for us to calculate the 30-day readmissions for a relatively large cohort of patients with AMI. Next, we will explore the types of AMI, according to the severity or procedures undertaken, to study the more significant impact of 30-day readmission rates by using the administrative data.

## 5. Conclusions

We have presented novel findings on an important tool—the LACE index with associated factors—to predict 30-day readmissions for the first time in South Korea. The LACE index can be computed without the aid of special software and does not require complex information such as community-specific rates of admission or economic statuses. Given its ease of use at the bedside, LACE is commonly used to risk-stratify hospitalized patients with medical illnesses. Therefore, we advise that focusing on the LACE index to predict the risk of a 30-day readmission will be critical for reducing the future readmission burden on the hospital care of patients with acute CVD. In addition, continued follow-up of AMI patients may also be needed to reduce the readmission risks among those directly discharged to their homes. The findings of this study will be valuable for healthcare managers in supporting them to implement policies on the use of the LACE index to easily predict the risk of early readmission and avoid unnecessary medical expenditure. The findings will assist the development of future interventions to predict 30-day readmissions and should be expanded by using national administrative data. Future research should be carried out with a prospective design, longer period, all the causes of 30-day readmissions and additional factors accounted for, to gain a better understanding of the association between 30-day readmissions and cost-effectiveness analyses by using the LACE index, and to demonstrate the lag effects of readmission rates on operating margins.

### Limitations

This study has certain limitations. First, the patients were selected from only one hospital in the metropolitan city, with information available only on 30-day readmissions to the same hospital where the patients were hospitalized for their index of admission, meaning our findings cannot be generalized, e.g., to other areas of Korea. Further work is needed to determine whether certain ICD-10 AMI codes represent 30-day readmissions that could be prevented through improved clinical-based care or healthcare systems. Moreover, our study was based on retrospective cohort data, meaning causation cannot be construed as there may be unnoticed confounding variables. Second, the cohort data were for patients hospitalized with AMI between 2015 and 2019 and, since then, changes were made to clinical guidelines, in particular, to the provision of acute services after a cardiovascular condition is diagnosed. However, these results remain valuable since, for the first time, reliable data on 30-day readmissions after the hospital discharge of patients with AMI have been described in South Korea.

## Figures and Tables

**Table 1:** Baseline Characteristics of 30-day Readmissions and Non-Readmissions in Patients Hospitalized with AMI.

Variables	Characteristics	30-Day Readmission				
		Yes (*n* = 205)		No (*n* = 3402)		*p*
		n	%	n	%	
Age (Years) ^†^ Median age, (IQR)		68.4 (12.9) 68 (58–77)		67.9 (11.2) 67 (51–68)		<0.001
Sex	Male	120	58.5	1928	56.7	<0.001
	Female	85	41.5	1474	43.3	
Residence	Seoul (capital area)	158	77.1	2163	63.6	<0.001
	Other Metropolitan cities	47	22.9	1239	36.4	
Health insurance	NHI	114	55.6	2796	82.2	0.008
	Medicare	86	42.0	486	14.3	
	Others	5	2.4	120	3.5	
Length of stay	<2	49	23.9	795	23.4	0.114
	3	81	39.5	908	26.7	
	4	22	10.7	708	20.8	
	5	24	11.7	622	18.3	
	6	18	8.8	221	6.5	
	≥7	11	5.4	148	4.4	
Admission route	ER	106	51.7	2191	64.4	<0.001
	OP	81	39.5	601	17.7	
	Transfer from another hospital	18	8.8	110	3.2	
Comorbidities by CCI	1	59	28.8	514	15.1	0.023
	2	68	33.2	1865	54.8	
	≥3	78	38.0	1023	30.1	
Discharge type	Normal	38	18.5	2988	87.8	0.021
	Other *	167	81.5	414	12.2	
Discharge destination	Home	44	21.5	2956	86.9	0.103
	Transfer to another hospital/facility	161	78.5	446	13.1	
LACE index score	0–4	42	20.5	838	24.6	<0.001
	5–9	71	34.6	1771	52.1	
	≥10	92	44.9	793	23.3	
Laboratory findings ^†^	SBP (mmHg)	125.1 (15.6)		120.8 (17.5)		0.191
	Hemoglobin, mg/dL	10.6 (9.3)		11.4 (9.8)		<0.001
	WBC, ×10^3^/μL	3.6 (1.1)		5.8 (3.0)		0.441
	Platelet, ×10^3^/μL	223.1 (99.8)		225.6 (111.8)		0.418
	Creatinine, mg/dL	1.65 (2.4)		1.2 (1.1)		0.541
	Potassium, mmol/L	3.9 (0.5)		4.04 (3.2)		0.842
	Sodium, mmol/L	137.2 (4.5)		139.5 (4.1)		0.691
	Estimated GFR (mL/min/m^2^)	39 (25.8)		41 (28)		0.511

^†^ = Mean (standard deviation); *n* (%) = number (percentage); *p*-value = Chi-squared test; NHI = national health insurance; CCI* = Charlson comorbidity index; ER = emergency route; WBC = white blood cells; GFR = glomerular filtration rate; OP = outpatient; * Home with support services, transferred to long-term care/another institution or left against medical advice.

**Table 2:** Distribution of LACE Score of Patients with AMI and 30-day Readmissions (*n* = 205).

Variables	Characteristics	LACE Index Score					
		0–4		5–9		≥10	
		*n*	%	*n*	%	*n*	%
		42	20.5	71	34.6	92	44.9
Age, years ^†^		68.2 (11.3)		67.5 (7.9)		68.9 (11.8)	
Sex	Male	27	64.3	39	54.9	54	58.7
	Female	15	35.7	32	45.1	38	41.3
Length of stay	<2	12	28.6	21	15.0	16	17.4
	3	10	23.8	34	47.9	37	40.2
	4	9	21.4	7	9.9	6	6.5
	5	6	14.3	8	11.3	10	10.9
	6	3	7.1	8	11.3	7	7.6
	≥7	2	4.8	3	4.2	6	6.5
Admission (Index of admission)	ER	30	71.4	35	49.3	51	55.4
	OP	7	16.7	21	29.6	44	47.8
	Transfer from another hospital	5	11.9	4	5.6	9	9.8
Comorbidities by CCI*	1	8	19.0	12	16.9	39	42.4
	2	15	35.7	15	21.1	38	41.3
	≥3	19	45.2	24	33.8	35	38.0
Emergency visits (past 6 months)	1	6	14.3	8	11.3	14	15.2
	2	4	9.5	14	19.7	12	13.0
	3	20	47.6	18	25.4	21	22.8
	4	12	28.6	31	43.7	45	48.9

^†^ = Mean (standard deviation); *n* (%) = number (percentage); CCI*= Charlson comorbidity index; ER = emergency route; OP = outpatient.

**Table 3:** Multivariate Logistic Regression Analysis for 30-day Readmission of Patients Hospitalized with AMI (*n* = 205).

Variables	Characteristics	30-Day Readmissions (Yes)			
		OR	95% CI		*p*
Age, years		1.78	1.54	2.05	<0.001
Sex	Male	1.07	1.06	1.07	<0.001
	Female	1.00			
Health insurance	NHI	1.00			
	Medicare	1.07	1.00	1.11	0.003
	Others	0.98	0.85	1.13	0.441
Admission route	ER	1.45	1.42	1.54	0.021
	OP	1.00			
Discharge type	Normal	1.00			
	Others *	2.89	1.49	5.60	<0.001
LACE index score	0–4	1.00			
	5–9	1.13	1.11	1.15	0.007
	≥10	2.71	1.03	4.37	0.010

OR = odds ratio; CI = confidence interval; AMI = acute myocardial infarction; NHI = national health insurance; ER = emergency visit; OP = outpatient visit; * Home with support services, transferred to long-term care/another institution or voluntary discharge.

## Data Availability

The data presented in this study are available on request from the corresponding author.

## References

[1] Hospital Readmissions—Healthcare.gov Glossary. https://www.healthcare.gov/glossary/hospital-readmissions/.

[2] The Hospital Readmissions Reduction (HRR) Program. https://www.cms.gov/Medicare/Medicare-Fee-for-Service-Payment/AcuteInpatientPPS/Readmissions-Reduction-Program.html.

[3] Reed G.W., Rossi J.E., Cannon C.P. (2017). Acute myocardial infarction. Lancet.

[4] Fingar K., Washington R. Trends in Hospital Readmissions for Four High-Volume Conditions, 2009–2013: Statistical Brief #196. The Healthcare Cost and Utilization Project Website. http://www.hcup-us.ahrq.gov/reports/statbriefs/sb196-Readmissions-Trends-High-Volume-Conditions.pdf.

[5] Pandey A., Golwala H., Hall H.M., Wang T.Y., Lu D., Xian Y., Chiswell K., Joynt K.E., Goyal A., Das S.R. (2017). Association of US Centers for Medicare and Medicaid Services Hospital 30-day risk-standardized readmission metric with care quality and outcomes after acute myocardial infarction: Findings from the National Cardiovascular Data Registry/acute coronary treatment and intervention outcomes network registry-get with the guidelines. JAMA Cardiol..

[6] Kristensen S.R., Bech M., Quentin W. (2015). A roadmap for comparing readmission policies with application to Denmark, England, Germany and the United States. Health Policy.

[7] Zhang Z., Qiu H., Li W., Chen Y. (2020). A stacking-based model for predicting 30-day all-cause hospital readmissions of patients with acute myocardial infarction. BMC Med Inform. Decis. Mak..

[8] Kwok C.S., Capers Q., Savage M., Gulati M., Potts J., Mohamed M.O., Nagaraja V., Patwala A., Heatlie G., Kontopantelis E. (2020). Unplanned hospital readmissions after acute myocardial infarction: A nationwide analysis of rates, trends, predictors and causes in the United States between 2010 and 2014. Coron. Artery Dis..

[9] Wang H., Zhao T., Wei X., Lu H., Lin X. (2019). The prevalence of 30-day readmission after acute myocardial infarction: A systematic review and meta-analysis. Clin. Cardiol..

[10] Shah R.U., de Lemos J.A., Wang T.Y., Chen A.Y., Thomas L., Sutton N.R., Fang J.C., Scirica B.M., Henry T.D., Granger C.B. (2016). Post-Hospital Outcomes of Patients With Acute Myocardial Infarction With Cardiogenic Shock. J. Am. Coll. Cardiol..

[11] Suter L.G., Li S.X., Grady J.N., Lin Z., Wang Y., Bhat K.R., Turkmani D., Spivack S.B., Lindenauer P.K., Merrill A.R. (2014). National patterns of risk standardized mortality and readmission after hospitalization for acute myocardial infarction, heart failure, and pneumonia: Update on publicly reported outcomes measures based on the 2013 release. J. Gen. Intern. Med..

[12] Lee S.E., Lee H.-Y., Cho H.J., Choe W.-S., Kim H., Choi J.O., Jeon E.-S., Kim M.-S., Kim J.-J., Hwang K.-K. (2017). Clinical characteristics, and outcome of acute heart failure in Korea: Results from the Korean Acute Heart Failure Registry (KorAHF). Korean Circ. J..

[13] Donzé J., Aujesky D., Williams D., Schnipper J.L. (2013). Potentially avoidable 30-day hospital readmissions in medical patients: Derivation and validation of a prediction model. JAMA Intern. Med..

[14] Billings J., Blunt I., Steventon A., Georghiou T., Lewis G., Bardsley M. (2012). Development of a predictive model to identify inpatients at risk of re-admission within 30 days of discharge (PARR-30). BMJ Open.

[15] Boyle J., Le Padellec R., Ireland D. Statewide validation of a patient admissions prediction tool. Proceedings of the 2010 Annual International Conference of the IEEE Engineering in Medicine and Biology.

[16] Damery S., Combes G. (2017). Evaluating the predictive strength of the LACE index in identifying patients at high risk of hospital readmission following an inpatient episode: A retrospective cohort study. BMJ Open.

[17] Hakim M.A., Garden F.L., Jennings M.D., Dobler C.C. (2018). Performance of the LACE index to predict 30-day hospital readmissions in patients with chronic obstructive pulmonary disease. Clin. Epidemiol..

[18] Ibrahim A.M., Koester C., Al-Akchar M., Tandan N., Regmi M., Bhattarai M., Al-Bast B., Kulkarni A., Robinson R. (2020). HOSPITAL Score, LACE Index and LACE+ Index as predictors of 30-day readmission in patients with heart failure. BMJ Evid. Based Med..

[19] Dobler C.C., Hakim M., Singh S., Jennings M., Waterer G., Garden F.L. (2020). Ability of the LACE index to predict 30-day hospital readmissions in patients with community-acquired pneumonia. ERJ Open Res..

[20] Wang H., Robinson R.D., Johnson C., Zenarosa N.R., Jayswal R.D., Keithley J., A Delaney K. (2014). Using the LACE index to predict hospital readmissions in congestive heart failure patients. BMC Cardiovasc. Disord..

[21] van Walraven C., Dhalla I.A., Bell C., Etchells E., Stiell I.G., Zarnke K., Austin P.C., Forster A.J. (2010). Derivation and validation of an index to predict early death or unplanned readmission after discharge from hospital to the community. Can. Med. Assoc. J..

[22] Regmi M.R., Bhattarai M., Parajuli P., Garcia O.E.L., Tandan N., Ferry N., Cheema A., Chami Y., Robinson R. (2020). Heart Failure with Preserved Ejection Fraction and 30-Day Readmission. Clin. Med. Res..

[23] Low L.L., Liu N., Wang S., Thumboo J., Ong M.E.H., Lee K.H. (2016). Predicting 30-Day Readmissions in an Asian Population: Building a Predictive Model by Incorporating Markers of Hospitalization Severity. PLoS ONE.

[24] Tan S.Y., Low L.L., Yang Y., Lee K.H. (2013). Applicability of a previously validated readmission predictive index in medical patients in Singapore: A retrospective study. BMC Health Serv. Res..

[25] Miller W.D., Nguyen K., Vangala S., Dowling E. (2018). Clinicians can independently predict 30-day hospital readmissions as well as the LACE index. BMC Heal. Serv. Res..

[26] Health Insurance Review & Assessment Service (2016). Study of Readmission Model Categorize and Standard Development.

[27] Labrosciano C., Air T., Tavella R., Beltrame J.F., Ranasinghe I. (2020). Readmissions following hospitalisations for cardiovascular disease: A scoping review of the Australian literature. Aust. Health Rev..

[28] Smith L.N., Makam A.N., Darden D., Mayo H., Das S.R., Halm E.A., Nguyen O.K. (2018). Acute myocardial infarction readmission risk prediction models: A systematic review of model performance. Circ. Cardiovasc. Qual. Outcomes.

[29] Au A.G., McAlister F., Bakal J.A., Ezekowitz J., Kaul P., van Walraven C. (2012). Predicting the risk of unplanned readmission or death within 30 days of discharge after a heart failure hospitalization. Am. Hear. J..

[30] Kim H.S., Kim Y., Kwon H. (2019). Health-related quality of life and readmission of patients with cardiovascular disease in South Korea. Perspect. Public Health.

[31] Rajaguru V., Han W., Kim T.H., Shin J., Lee S.G. (2022). LACE Index to Predict the High Risk of 30-Day Readmission: A Systematic Review and Meta-Analysis. J. Pers. Med..

